# Comparison of HIIT and MICT and further detraining on metabolic syndrome and asprosin signaling pathway in metabolic syndrome model of rats

**DOI:** 10.1038/s41598-024-61842-5

**Published:** 2024-05-17

**Authors:** Hiwa Ahmed Rahim, Arsalan Damirchi, Parvin Babaei

**Affiliations:** 1 Department of Exercise Physiology, Faculty of Sport Sciences, University of Guilan, Rasht, Iran; 2 College of Physical Education and Sports Sciences, University of Halabja, Halabja, Iraq; 3 Neuroscience Research Center, Trauma Institute, Guilan University of Medical Sciences, Rasht, Iran; 4 Cellular & Molecular Research Center, School of Medicine, Guilan University of Medical Sciences, Rasht, Iran; 5 Department of Physiology, School of Medicine, Guilan University of Medical Sciences, Rasht, Iran

**Keywords:** High-intensity interval training (HIIT), (MICT) moderate-intensity continuous training, Metabolic syndrome, Diabetes, Asprosin, Insulin resistance, Physiology, Diseases, Medical research, Risk factors

## Abstract

Physical activity promotes various metabolic benefits by balancing pro and anti-inflammatory adipokines. Recent studies suggest that asprosin might be involved in progression of metabolic syndrome (MetS), however, the underlying mechanisms have not been understood yet. This study aimed to evaluate the effects of high-intensity interval training (HIIT), moderate-intensity continuous training (MICT), and further detraining on MetS indices, insulin resistance, serum and the liver levels of asprosin, and AMP-activated protein kinase (AMPK) pathway in menopause-induced MetS model of rats. A total of 64 Wistar rats were used in this study and divided into eight groups: Sham1, OVX1 (ovariectomized), Sham2, OVX2, OVX + HIIT, OVX + MICT, OVX + HIIT + Det (detraining), and OVX + MICT + Det. Animals performed the protocols, and then serum concentrations of asprosin, TNF-α, insulin, fasting blood glucose, and lipid profiles (TC, LDL, TG, and HDL) were assessed. Additionally, the liver expression of asprosin, AMPK, and P-AMPK was measured by western blotting. Both HIIT and MICT caused a significant decrease in weight, waist circumference, BMI (*P* = 0.001), and serum levels of glucose, insulin, asprosin (*P* = 0.001), triglyceride, total cholesterol, low-density lipoprotein (LDL), and TNF-α (*P* = 0.001), but an increase in the liver AMPK, P-AMPK, and P-AMPK/AMPK (*P* = 0.001), compared with OVX2 noexercised group. MICT was superior to HIIT in reducing serum asprosin, TNF-a, TG, LDL (*P* = 0.001), insulin, fasting blood glucose, HOMA-IR, and QUEKI index (*P* = 0.001), but an increase in the liver AMPK, and p-AMPK (*P* = 0.001). Although after two months of de-training almost all indices returned to the pre exercise values (*P* < 0.05). The findings suggest that MICT effectively alleviates MetS induced by menopause, at least partly through the activation of liver signaling of P-AMPK and the reduction of asprosin and TNF-α. These results have practical implications for the development of exercise interventions targeting MetS in menopausal individuals, emphasizing the potential benefits of MICT in mitigating MetS-related complications.

## Introduction

Menopause is an important period of women's life between the ages of 45–55 and is associated with estrogen deficiency and further consequences including weight gain, central adiposity, dyslipidemia, and high blood glucose^1^, which result in metabolic syndrome (MetS)^2^. Metabolic syndrome is one of the major public health problem^3^, predisposing individuals toward insulin resistance (IR), type II diabetes, and cardiovascular diseases (CVD)^4^.

Adipose tissue, as an active endocrine organ secretes various proteins known as adipokines^5^ which are categorized as pro and anti-inflammatory cytokines and play crucial roles in the regulation of metabolism and inflammation^6^. One of the newly discovered adipokines with pro-inflammatory functions is asprosin^7^, playing a significant role in insulin resistance, type 2 diabetes, and obesity^8^. It has been assumed that asprosin increases circulating levels of TNFα, and IL-6^9^, and induces apoptosis and inflammation^10^. Despite recent investigations on asprosin, the mechanisms involving asprosin functions in MetS have not been elucidated completely.

One of the main strategies to combat MetS and prevent its progression toward serious metabolic disorders is physical exercise^11^. Exercise training exerts molecular and systemic changes in the body to decrease fat mass, improve both skeletal muscle functions and insulin sensitivity^12,13^. It also promotes the production of anti-inflammatory cytokines^14^, while reduces the pro-inflammatory ones^15^. These effects make exercise interventions valuable strategy for preventing insulin resistance, and type 2 diabetes^16^. However, there is controversy about the volume, intensity, frequency, and modality of exercise training for a specific target^17^. For example, high-intensity interval training (HIIT), which defines by multiple repetitions^4–10^, brief bouts (20–300 s) of high-intensity exercise (80–100% peak heart rate, interspersed with rest^18^, has been reported to exert great beneficial cardiovascular benefits^19^, by alleviating inflammatory markers and lipids^20^, also stimulating mitochondrial biogenesis, and angiogenesis^21^.

On the other hand, moderate-intensity continuous training (MICT) is defined by continuous bout of moderate-intensity aerobic activity at a steady state of set duration (typically between 20 and 60 min)^22^. MICT has been shown to improve cardiovascular fitness, enhance aerobic capacity and lowers blood pressure^23,24^. Additionally, MICT has been reported to improve insulin sensitivity, glucose metabolism, and reduces waist circumference^25,26^, and visceral adipose tissue^14^.

Despite the efficacy of both HIIT and MICT in metabolic health^18,24,27^, maintenance of their metabotropic adaptations are challengeable. Most of studies on detraining effects which focused on musculoskeletal outcomes reported severe negative effects after 8 weeks of exercise cessation^28,29^. Which protocol is superior in inducing long lasting metabolic adaptations after detraining in menopause induced MetS model, has not been understood yet. Therefore, this study aimed to compare the effects of 12-week HIIT and MICT, and 2 months detraining on MetS indices pointing to the inflammatory cytokines of asprosin, TNF-a, and molecular signaling pathway of P-AMPK/ AMPK, in ovariectomized rats with MetS.

## Results

One month following the ovariectomy surgery, data revealed significant increase in body weight (*P* = 0.001), BMI (*P* = 0.002), waist circumference (WC) (*P* = 0.001), low-density lipoprotein (LDL) cholesterol (*P* = 0.002), triglycerides (TG) (*P* = 0.001), total cholesterol (TC) (*P* = 0.001), fasting blood glucose (*P* = 0.001), insulin (*P* = 0.001), and homeostatic model assessment of insulin resistance (HOMA-IR) and QUEKI (*P* = 0.001), but a significant reduction in high-density lipoprotein cholesterol (HDL-C) (*P* = 0.003, Table 1).

**Table 1:** Comparison of (mean ± SD) components of MetS between Ovx1 and sham1 group one month after ovariectomized.

Variable	Groups		*P* value^a^
	Sham1	Ovx1	
Body weight (g)	243.50 ± 6.611	259.50 ± 8.701	0.001*
BMI (kg/m^2^)	0.503 ± 0.04	0.534 ± 0.06	0.002*
WC (cm)	14.50 ± 0.53	15.62 ± 0.74	0.001*
Food Intake (g)	16.12 ± 0.99	17.00 ± 1.69	0.128
TC (mg/dL)	50/75 ± 1/66	60/87 ± 2/03	0.001*
TG (mg/dL)	41/12 ± 2/94	49/00 ± 2/0	0.001*
LDL (mg/dL)	60/12 ± 2/23	65/37 ± 3/06	0.002*
HDL (mg/dL)	36/00 ± 0/00	32/25 ± 2/25	0.003*
Insulin (μU/ml)	3.58 ± 0.64	4.28 ± 0.32	0.001*
Glucose (mg/dL)	143/12 ± 26/37	217/00 ± 26/84	0.001*
HOMA-IR	1/26 ± 0/32	2/29 ± 0/24	0.001*
QUEKI	0.33 ± 0.005	0.37 ± 0.013	0.001*

*BMI* body mass index, *WC* waist circumference, *HDL-C* high-density lipoprotein cholesterol, *LDL-C* low-density lipoprotein cholesterol, *ovx*, ovariectomy, *TG* Triacylglyceride. Values are reported as the mean ± SE for eight animals per group.

^a^*P*: Statistical analysis was done by independent t-test; *P < 0.05 versus (sham) sham-operated group.

### Effect of HIIT and MICT and further detraining on body weight, BMI, and WC

**Figure 1:**
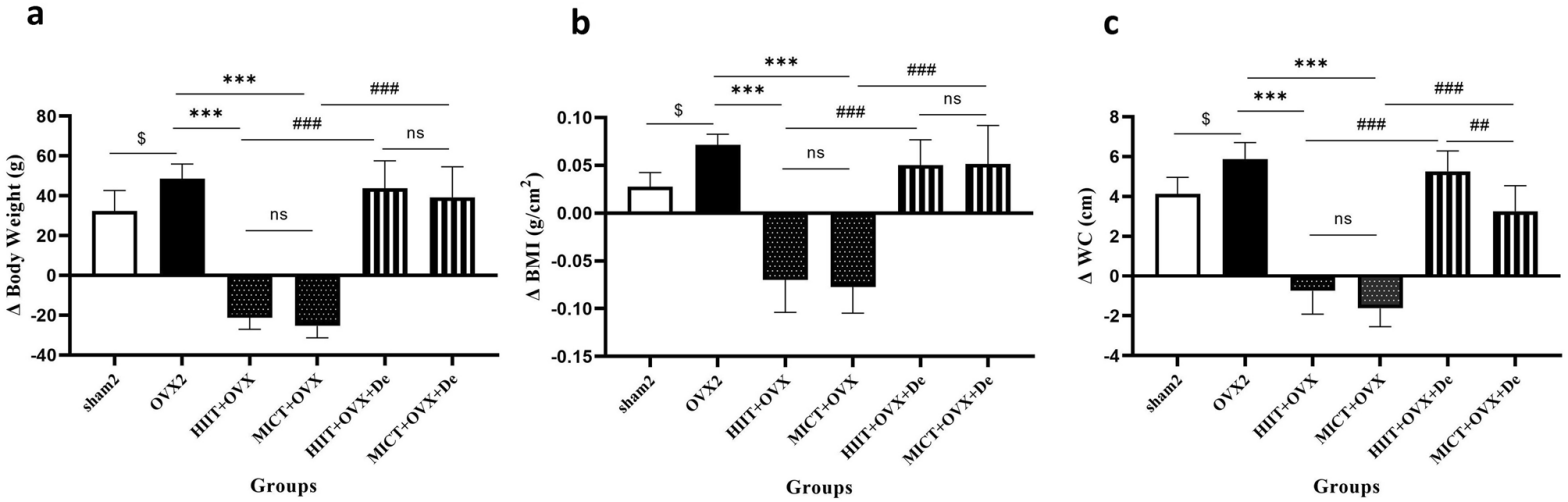
The effect of 12 week HIIT and MICT training on (**a**) body weight, (**b**) BMI, body mass index; and (**c**) WC, waist circumference. Ovx2, Three-month ovariectomized group; Ovx + HIIT, ovariectomized with high-intensity interval training group; Ovx + MICT, ovariectomized with moderate-intensity continuous training group; OVX + MICT + De, ovariectomized with high-intensity interval training for two month detraining group, OVX + MICT + De, ovariectomized with moderate-intensity continuous training group sham, sham-operated group; One-way analysis of variance test followed by Tukey's post hoc test. $*P* < 0.0001 versus OVX2 and sham2 group. *P < 0.0001 versus Ovx + HIIT and Ovx + MICT group, ≠ *P* < 0.0001 versus OVX + HIIT + De and OVX + MICT + De.

Both groups of MICT + OVX and HIIT + OVX exhibited significant reductions in weight (23%, 27%), BMI (37%, 19%) and waist circumference (23% and 32%) (*P* = 0.001) compared with the Ovx2. After detraining these three parameters reversed to the pre training value in HIIT + OVX + De and MICT + OVX + De respectively (Fig. 1a–c).

### Effect of HIIT and MICT and further detraining on Insulin, Glucose, HOMA-IR, QUEKI index

**Figure 2:**
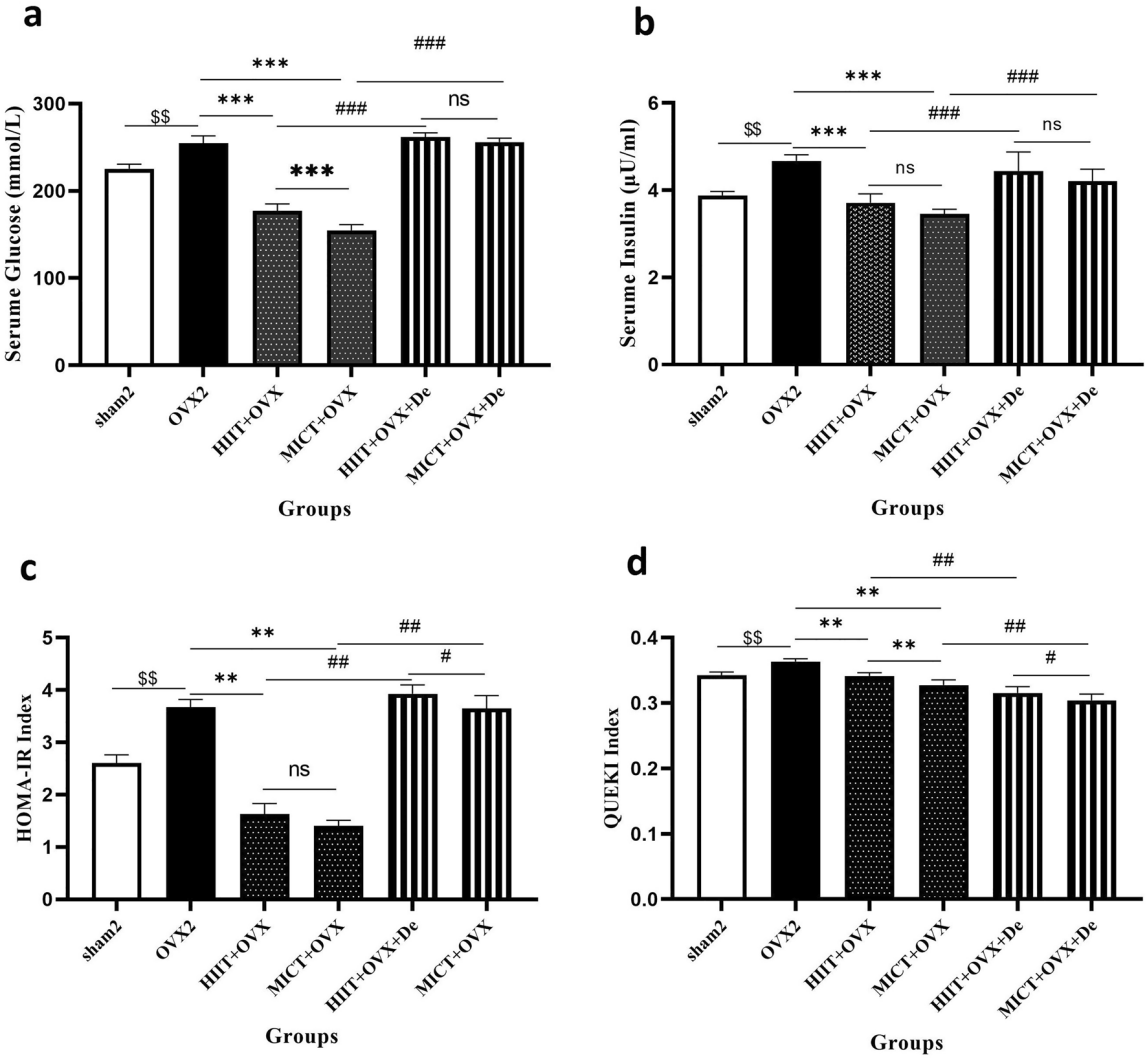
The effect of 12 week HIIT and MICT training on (**a**) Glucose, (**b**) Insulin; (**c**) HOMA-IR and (d) QUEKI Index. Ovx2, Three-month ovariectomized group; Ovx + HIIT, ovariectomized with high-intensity interval training group; Ovx + MICT, ovariectomized with moderate-intensity continuous training group; OVX + HIIT + De, ovariectomy with high-intensity interval training detraining group; OVX + MICT + De, ovariectomy with high-intensity interval training detraining group. sham, sham-operated group; One-way analysis of variance test followed by Tukey's post hoc test. $*P* < 0.0001 versus OVX2 and sham2 group. *P < 0.0001 versus Ovx + HIIT and Ovx + MICT group, ≠ *P* < 0.0001 versus OVX + HIIT + De and OVX + MICT + De.

Significant increase was found in insulin, glucose, HOMA-IR and QUEKI index (*P* = 0.001) in OVX2 compared to the sham2 group, which then were reduced by − 20%, − 30%, − 55% and − 6% in HIIT + OVX and − 26%, − 39%, − 61% and − 8% in MICT + OVX. In addition, detraining in both groups of HIIT + OVX + De and MICT + OVX + De reversed the alteration made during trainings (*P* = 0.001) (Fig. 2a–d).

### Effect of HIIT and MICT and further detraining on Lipid profile: TC, TG, LDL and HDL

Total cholesterol (TC) was elevated in OVX2 by 43% compared with sham2, after engaging in HIIT reduced by − 36% and − 4%, in MICT + OVX groups (*P* = 0.001). Similarly, TG and LDL decreased by 9%, *P* = 0.001), (− 36%, *P* = 0.001) in HIIT and (− 17%, − 42%, *P* = 0.001), in MICT + OVX compared to OVX2 groups, respectively. However, the MICT + OVX group demonstrated a greater magnitude of reduction in these parameters compared to the HIIT + OVX group. Also, in HIIT + OVX + De and MICT + OVX + De groups, a significant increase in these parameters was observed again after 2 months of detraining.

Furthermore, low-density lipoprotein (LDL) levels were significantly reduced by (− 0.51%, *P* = 0.001), (− 26%, *P* = 0.001) and (− 16%, *p* = 0.001), (− 38%, *P* = 0.001) in both the HIIT + OVX and MICT + OVX groups compared to the Sham2 and OVX2 groups, respectively. However, there was significant by 35% difference between Sham2 and OVX2 groups. Also, OVX + MICT group showed more reduction in LDL level compared with the OVX + HIIT group (43% *P* = 0.001).

**Figure 3:**
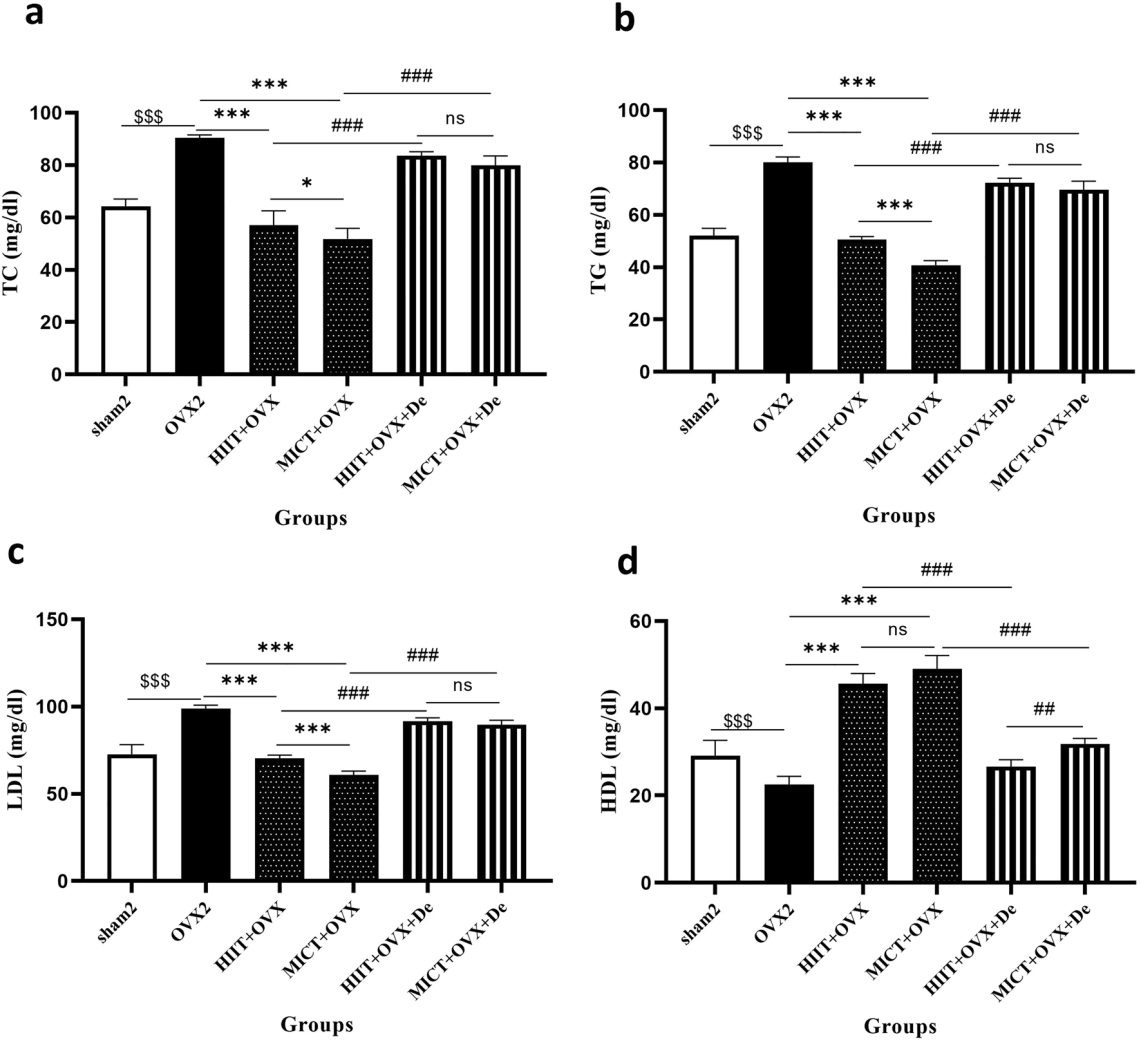
The effect of 12 week HIIT and MICT training on (**a**) Total cholesterol (TC), (**b**) Triacylglyceride (TG), (**c**) LDL-C, low-density lipoprotein cholesterol (LDL) and (**d**) HDL-C, high-density lipoprotein cholesterol; Ovx2, Three-month ovariectomized group; Ovx + HIIT, ovariectomized with high-intensity interval training group; Ovx + MICT, ovariectomized with moderate-intensity continuous training group; OVX + HIIT + De, ovariectomy with high-intensity interval training detraining group; OVX + MICT + De, ovariectomy with high-intensity interval training detraining group. Sham, sham-operated group; TC, total cholesterol; TG, Triacylglyceride One-way analysis of variance test followed by Tukey's post hoc test. Values are shown as the mean ± SE for eight animals per group. $*P* < 0.0001 versus OVX2 and sham2 group. *P < 0.0001 versus Ovx + HIIT and Ovx + MICT group, ≠ *P* < 0.0001 versus OVX + MICT + De and OVX + MICT + De.

HDL was significantly decreased in Sham2 and OVX2 groups, (− 22%, *P* = 0.001) compared with the HIIT + OVX and MICT + OVX groups, but elevated by 102% in HIIT + OVX and 117%, MICT + OVX groups (*P* = 0.001). Also, in HIIT + OVX + De and MICT + OVX + De groups, a significant decreased in these HDL was observed again after 2 months of detraining. However, the MICT + OVX + De group demonstrated a greater magnitude of reduction in these parameters compared to the HIIT + OVX + De group (Fig. 3a–d).

### Effect of HIIT and MICT on MetS Z-score

**Figure 4:**
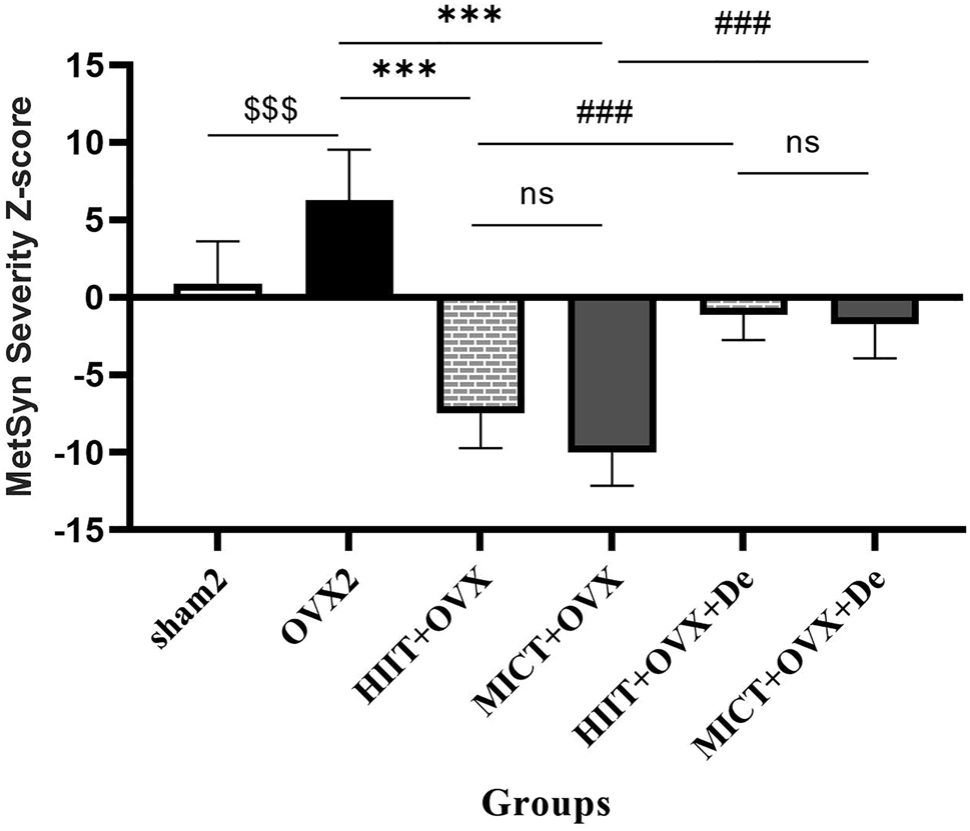
Change in MetS Z-score after interventions. One-way analysis of variance test followed by Tukey’s post hoc test revealed significance difference. $*P* < 0.0001 versus OVX2 and sham2 group. *P < 0.0001 versus Ovx + HIIT and Ovx + MICT group, ≠ *P* < 0.0001 versus OVX + MICT + De and OVX + MICT + De. Ovx2, Three-month ovariectomy group; Ovx + MICT, ovariectomy with high-intensity interval training group; Ovx + MICT, ovariectomy with moderate-intensity continuous training group; sham, sham-operated group; OVX + HIIT + De, ovariectomy with high-intensity interval training detraining group; OVX + MICT + De, ovariectomy with high-intensity interval training detraining group.

Finally, one-way ANOVA test showed a significant difference in MetS Z-score between groups (Fig. 4), in a way that it was significantly decreased in the treated groups compared with the OVX2 group (*P* = 0.001). Figure 4 shows the MetS Z-score, which was successfully decreased in the OVX + HIIT and OVX + MICT rats compared with the sham2 group, however, after the detraining MetS-Z-score reversed in both groups.

### Effect of HIIT and MICT and further detraining on serum level of asprosin

**Figure 5:**
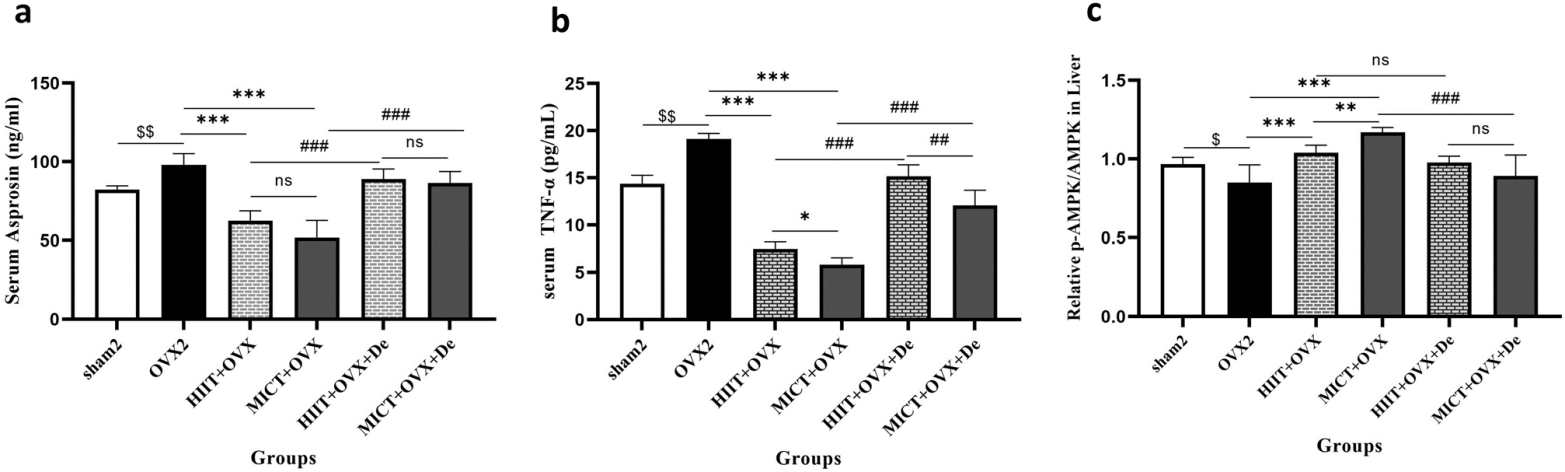
The effect of 12 week HIIT and MICT training on (**a**) Serum Asprosin (**b**) Serum TNF-a, and (**c**) p-AMPK/AMPK Liver. Values are reported as the mean ± SE for eight animals per group. Ovx2, Three-month ovariectomy group; Ovx + MICT, ovariectomy with high-intensity interval training group; Ovx + MICT, ovariectomy with moderate-intensity continuous training group; sham, sham-operated group; OVX + HIIT + De, ovariectomy with high-intensity interval training detraining group; OVX + MICT + De, ovariectomy with high-intensity interval training detraining group; TNF-a, tumor necrosis factor; AMPK, AMP-activated protein kinase. $*P* < 0.0001 versus OVX2 and sham2 group. *P < 0.0001 versus Ovx + HIIT and Ovx + MICT group, ≠ *P* < 0.0001 versus OVX + MICT + De and OVX + MICT + De.

After 12 weeks of intervention, ANOVA one way and Tukey test pairwise comparisons showed significant difference in serum asprosin level between sham2 and OVX2 (*P* = 0.001). While the level of asprosin in the OVX + HIIT and OVX + MICT groups was significantly decreased by − 36%, *p* = 0.001, and − 47%, (*P* = 0.001) compared with OVX2. Also, the OVX + MICT group showed more reduction in serum Asprosin level compared with the OVX + HIIT group (30% *P* = 0.001) (Fig. 5a).

### Effect of HIIT and MICT and further detraining on serum level of TNF-α

After 12 weeks of intervention, one-way ANOVA and Tukey's pairwise comparisons revealed a significant increase in the serum level of TNF-α in the OVX2 and sham2 groups (33%, *P* = 0.001). This increase was subsequently reduced by 70% in the OVX + MICT group and by 60% in the OVX + HIIT group (*P* = 0.001). Furthermore, in the HIIT + OVX + De and MICT + OVX + De groups, a significant increase in this parameter was observed again after 2 months of detraining. Notably, the HIIT + OVX group exhibited a greater magnitude of increase in this parameter compared to the MICT + OVX group (31%, *P* = 0.001) (Fig. 5b).

### Effect of HIIT and MICT and further detraining on serum level of p-AMPK/AMPK Liver

Significant decrease was found in p-AMPK/AMPK liver (*P* = 0.001) in OVX2 compared to the sham2 group. Also, after 12 weeks of intervention, one-way ANOVA and Tukey's pairwise comparisons revealed a significant increase of p-AMPK/AMPK liver in the OVX + HIIT and OVX + MICT groups compared with OVX2 group (*P* < 0.05). Additionally, in the HIIT + OVX + De and MICT + OVX + De groups, no significant changes in the above parameters was observed after 2 months of detraining (Fig. 5c).

### Effect of HIIT and MICT and further detraining on asprosin, p-AMPK, AMPK in the liver

**Figure 6:**
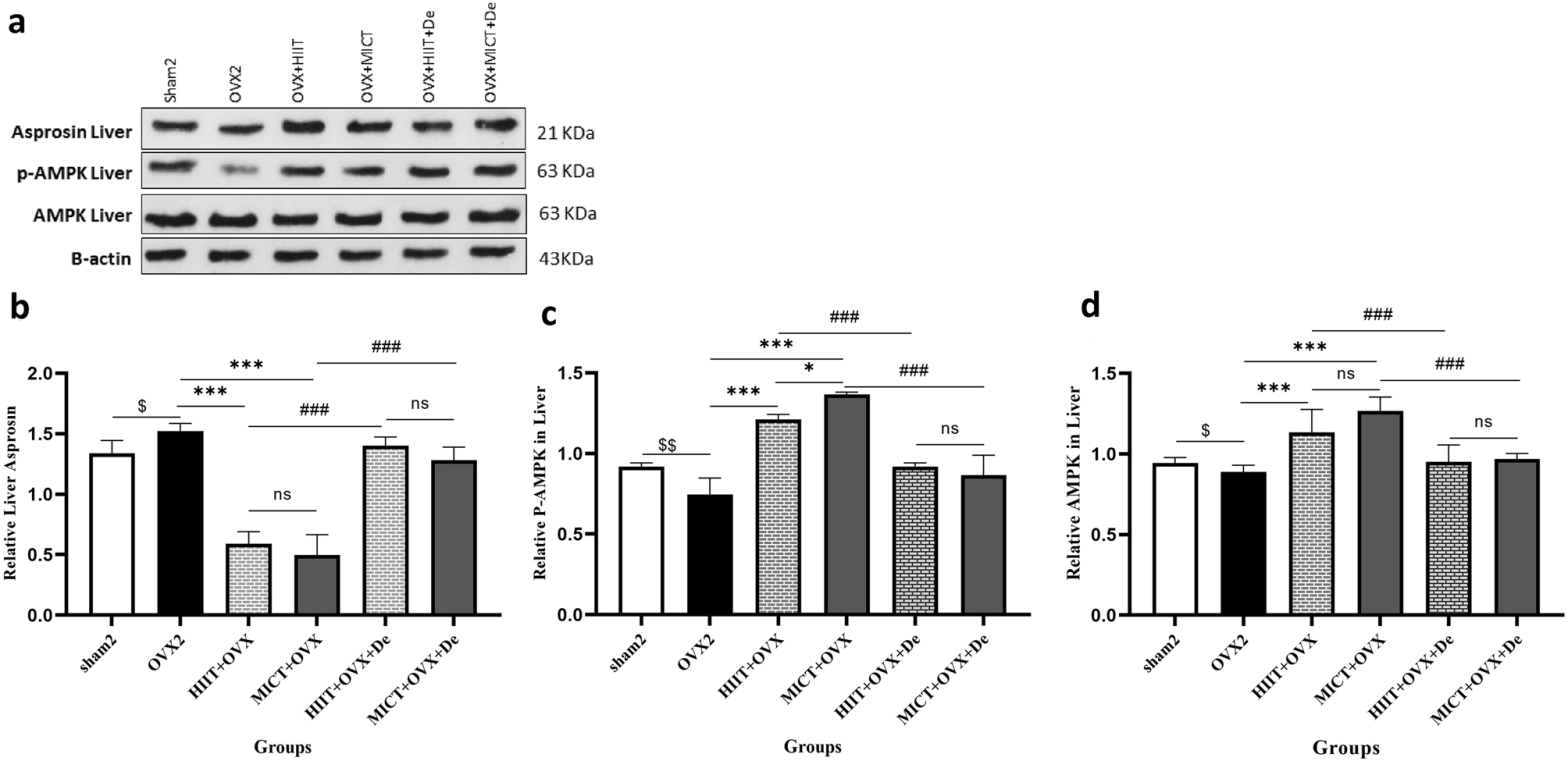
The effect of 12 week HIIT and MICT training on (**a**) B-actin (**b**) Asprosin Liver, (**c**) p-AMPK and (**d**) AMPK Liver. Values are reported as the mean ± SE for eight animals per group. Ovx2, Three-month ovariectomy group; Ovx + MICT, ovariectomy with high-intensity interval training group; Ovx + MICT, ovariectomy with moderate-intensity continuous training group; sham, sham-operated group; OVX + HIIT + De, ovariectomy with high-intensity interval training detraining group; OVX + MICT + De, ovariectomy with high-intensity interval training detraining group; TNF-a, tumor necrosis factor; AMPK, AMP-activated protein kinase. $*P* < 0.0001 versus OVX2 and sham2 group. *P < 0.0001 versus Ovx + HIIT and Ovx + MICT group, ≠ *P* < 0.0001 versus OVX + MICT + De and OVX + MICT + De. Original blots are available in supplementary files.

Western blot data in Fig. 6 shows a significant decrease in asprosin level in both OVX + HIIT and OVX + MICT groups compared with OVX2 by 102%, and 117%, *P* = 0.001) respectively) The expression of AMPK, as well as the activated form of p-AMPK, were significantly increased in the liver of the OVX + HIIT and OVX + MICT groups compared with OVX2 group (*P* < 0.05). However, the MICT + OVX group demonstrated a greater magnitude of increase in p-AMPK compared to the HIIT + OVX group. Additionally, in the HIIT + OVX + De and MICT + OVX + De groups, a significant increase in the above parameters was observed after 2 months of detraining, although it did not reach statistical significance (Fig. 6a–d).

## Discussion

The results of the present study are in line with the previous findings^30–32^ confirmed that ovariectomy leads in MetS by increasing body weight, body mass index, WC, FBG, lipids profile, serum insulin, asprosin and TNF-α one month after the surgery. In addition, we showed that 12-week HIIT and MICT efficiently reduced weight, BMI, WC, FBG, insulin resistance, and lipid profile in parallel with reduction in serum levels of asprosin, TNF-α, and elevation in AMPK and P-AMPK/AMPK in the liver. However, the MICT protocol was remarkably superior in alleviating most indices. Another key finding from the present study is that detraining for a 8- week leads to reversion of some indices including body weight, BMI, WC. FBG, lipids profile, serum asprosin, TNF-a, and P-AMPK.

Asprosin, a novel hormone released by white adipose tissue, has been found to have a significant association with glucose dis-homeostasis^33^, and MetS^34,35^. Finding of elevation in asprosin in menopause-induced MetS model in our study confirms the results from Alan et al., 2018 in women with polycystic ovary syndrome, and Naiemian et al.^36^, in diabetic patients.

Asprosin binds with olfactory receptor 734 (Olfr734), and leads to glucose release via cAMP and protein kinase (PKA) in the liver^7,33^, and finally induces insulin resistance^7^.

Reduction in the protein level of AMPK and P-AMPK in the liver, as well as P-AMPK/ AMPK ratio in the ovariectomized group reflects inhibitory action of this pathway by MetS status^37^. Therefore, elevation in asprosin in MetS impairs insulin secretion by down regulating P-AMPK/ AMPK signaling pathways^38^. Moreover elevation in Asprosin in our study was parallel with TNF-α, indicating that asprosin is a pro inflammatory adipokine to exert insulin resistance and dyslipidemia in menopause -induced MetS model.

Asprosin activates toll like receptors pathway and reduces skeletal muscle insulin sensitivity via elevation in inflammatory cascades^39^, and serum TNF-α^40^. Therefore, increased asprosin might be a good biomarker to reflect visceral fats accumulation, and insulin signaling impairment^9,10,41^.

In the next part of our experiment, our findings showed that 12-week of HIIT and MICT efficiently reduced weight, BMI, WC, FBG, insulin resistance, and lipid profile in parallel with reduction in serum levels of asprosin, TNF-a, and elevation in AMPK and P-AMPK/AMPK in the liver, with superiority of MICT in decreasing BMI by 15%, WC 14%, FBG 28%, insulin resistance 11%, and lipid profile by 26%.

Although, both HIIT and MICT training produce different physiological adaptations^42,43^, MICT training exerts higher rate of fat oxidation^44^ and lower risk of injury^45^, and is more suitable for patients with inflammatory status such a coronary disease, diabetes and obesity^46,47^, in contradictory with HIIT^48,49^.

Despite beneficial effects of exercise training on body weight reduction, there is evidence showing lack of weight reduction after exercise, especially in menopausal women. Several studies have revealed the importance of aimed at preventing metabolic syndrome in postmenopausal women. The present study confirmed this with the observation of a significant decline in body weight, BMI, WC, FBG, insulin resistance, and lipid profile in parallel with reduction in serum levels of asprosin, TNF-a, levels.

Due to the differences in duration and intensity of training between HIIT and MICT, the metabolic responses to these two exercise methods differ. A sustained training session as it is in MICT, is likely to be associated with lower energy expenditure, but it leads to increased lipolysis, resulting in the release of free fatty acids and subsequent fat oxidation. In this type of exercise, fat becomes the dominant fuel source. On the other hand, HIIT stimulates hormones such as cortisol, catecholamine’s, and growth hormones, all of which play an important role in improving body composition and promoting lipolysis^53^.

Despite all beneficial effects of HIIT in terms of weight loss and cardiovascular fitness^54^, it increases stress oxidative^55^, and leads to cellular damage and metabolic dysfunction, particularly in subjects with MetS. Meanwhile, there is a muscle hypoxia during HIIT, due to higher metabolic demands of active muscles^56^, which is followed by production of mitochondrial reactive oxygen species^57,58^.

Considering the fact that exercise as an important epigenetic factor, which influences on tremendous genes, transcriptional factors and regulatory enzymes, it remains to be elucidating the relationship between intensity, duration and frequency of training on epigenomes plasticity in various statuses of subjects^59^.

In conclusion, MICT, being performed at a moderate intensity, represented a more favorable impact on insulin sensitivity and inflammation in our study, partly via decrease in serum asprosin levels. This indicates that increased energy expenditure, and reduced fat mass in MICT group might lead to a reduction in asprosin and TNF-α, secretion from adipose tissue^33^, and thus, improves insulin sensitivity, at least partly via activation of the liver P-AMPK/ AMPK.

Finally, two months of detraining demonstrated that improvement in MetS indices such as HOMA-IR, QUEKI index, lipid profile and reduction in weight, BMI, WC, in both groups of HIIT + OVX and MICT + OVX returned almost to the pre training status, nevertheless the values remained higher than before training. To explain this phenomenon, a compensatory effect in fat metabolism after the interruption of exercise should be considered, since previous studies suggest that there is an increase in lipogenesis after the cessation of exercise^60^. Interestingly, TG, insulin, and glucose in both groups returned to lower values than baseline; particularly in HIIT group. Our finding is in agreement with the study by Damirchi 2014, who showed that a period of 4–8 weeks detraining is sufficient to lose metabolic and functional adaptations in subjects with MetS^61^. Therefore, long lasting exercise programs with moderate intensity may offer more functional protection.

Our study is the first to demonstrate reduction in the liver and serum asprosin levels following 12 weeks of HIIT and MICT in a menopause-induced MetS of rats. However, some limitations should be considered in future research, such as the assessment of oxidative stress pathways and other asprosin-related pathways (e.g., PKA, Akt, and PGC-1).

From clinical importance of view, it seems that serum asprosin could be a biomarker for MetS. Furthermore, MICT protocol may hold promise as a therapeutic approach for treating metabolic syndrome.

## Conclusions

Our findings suggest that 8 weeks of MICT training reversibly alleviates metabolic syndrome indices at least partly via reducing hepatic, and serum asprosin levels, and elevating P-AMPK/AMPK ratio, which lasts not longer than month.

## Materials and methods

### Animal care

Sixty-four female Wistar rats, aged 2–3 months, and body weight of 200 ± 20 g were provided from the Laboratory Animal Breeding Center of the Faculty of Pharmacy and kept four per cage in a standard environment (temperature of 22 ± 2 °C, and a 12-h light/dark cycle) in the Animal Lab of sports physiology department, and had ad libitum access to food and water. This study used double-blind, simple random selection with homogeneity as its sampling methodology. The Ethics Committee of Sport Sciences Research Institute of Iran approved the study protocol, and all methods used in this study are reported in accordance with ARRIVE guidelines (IR.GUILAN.REC.1401. 024). All methods were performed in accordance with the relevant guidelines and regulations.

### Ovariectomy surgery

At the age of 10 weeks, female Wistar rats were anesthetized by intraperitoneal injection of ketamine (50 mg/kg) and xylazine (5 mg/kg), in a ratio of 4:1 and underwent ovariectomy or sham operation after deep anesthesia and painless procedure. The procedure was performed through a small abdominal incision through which the ovaries were closed and removed bilaterally. The uterine horns were closed and the uterus remained healthy. After the surgery, the animals were kept in a standard recovery condition. For sham surgery, the animals underwent anesthesia and surgery similar to the ovariectomized groups with no removal of ovaries.

One month after the ovariectomy surgery, animals which demonstrated the metabolic syndrome characteristics were introduced to the study^30^.

### Animal groups

**Figure 7:**
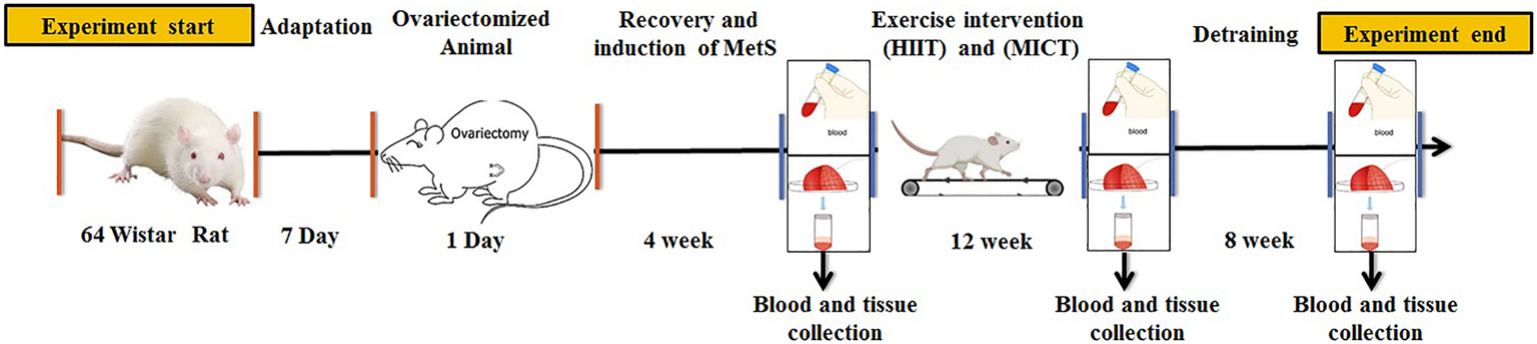
Schematic representation of study design and timeline. The timeline of experiments starting at 0 week point to 32 week end point analysis has been represented. After 7 day adaptation the rats was ovariectomized. After 4 weeks of recovery, the animals were ready for intervention and before the intervention, 2 groups were sacrificed and serum and liver tissue samples were collected (pre-test). 12 weeks of (HIIT) and (MICT) exercises were performed, and after 48 h, the rats were sacrificed and serum and liver tissue samples were collected. Finally, 2 groups were subjected to a detraining for 8 weeks, and then serum and liver tissue samples were collected and end point analysis was done by western blot and immunohistochemistry techniques.

The ovariectomized rats (OVX) were randomly assigned to the following: OVX + HIIT, OVX + MICT, OVX + treadmill off (stress control), OVX + HIIT + Det (detraining) and OVX + MICT + Det which were detained for 2 months. One extra ovariectomy and sham were sacrificed one month after the surgery for measuring MetS parameters confirming the establishment of MetS. The second sham group kept their routine till the end of the experiment. The trained groups performed running drills on a rodent treadmill with five lanes (DSI-580; Danesh Salar Iranian). Figure 7 shows the experimental design in detail.

### Acclimatization stage and VO2max measurement:

All animals were subjected to one week of acclimatization sessions consisting of placing on a rodent treadmill (10–15 min, for five times), with a zero-degree gradient, and speed of 8–10 m/min.

Initially, rats were placed on a treadmill with a 20-degree incline and warmed up by running at a speed of 6 m/min for 5 min, and then speed was increased by 2 m/min every 2 min until the rats reached the point of exhaustion. Exhaustion was defined as the moment when the rats remained on the lanes and stimulated with a weak electric shock to continue running for more than 10 s, and the time, speed, and distance traveled were recorded. The highest running speed achieved at the time of VO2max or VO2peak was considered as the maximum speed^41,62^.

### Intervention (HIIT) and (MICT) protocol

We modified the protocol used by Hofstad et al.^63^, consisting of 12 weeks (5 sessions per week) running on a treadmill with a 25-degree incline. Each session consisted of 10 bouts of 4-min activity with an intensity equal to 85–90% of VO2 max, and also active rest periods of 2 min with an intensity of 45–50% of VO2 max. Treadmill speed was incrementally raised from 17 to 26 met/min until the 10th week, and then remained unchanged. The MICT protocol was performed with an intensity equal to 65–70% of VO2 max, and similar distance covered in HIIT protocol. Also, 10 min of warming up and 5 min of cooling down with low intensity were performed at the beginning and the end of each training session^63^.

#### Intervention

High-Intensity Interval Training (HIIT) Protocol

- Duration: The HIIT protocol spanned 12 weeks, with 5 sessions per week.
- Treadmill Running: Participants engaged in treadmill running with a 25-degree incline.
- Session Structure: Each session comprised 10 bouts of 4-min activity at an intensity of 85–90% of VO2 max, interspersed with 2-min active rest periods at an intensity of 45–50% of VO2 max.
- Treadmill Speed: The treadmill speed was progressively increased from 17 met/min to 26 met/min until the 10th week, after which it remained constant.
- Warm-Up and Cool-Down: A 10-min warm-up and 5-min cool-down with low intensity were performed at the beginning and end of each training session^63^.

Moderate-Intensity Continuous Training (MICT) Protocol

- Intensity: The MICT protocol was conducted at an intensity equivalent to 65–70% of VO2 max, covering a similar distance as the HIIT protocol.
- Duration: Similar to the HIIT protocol, the MICT protocol extended over 12 weeks, with 5 sessions per week.
- Treadmill Running: Participants were engaged in treadmill running, following a consistent intensity throughout the sessions.
- Warm-Up and Cool-Down: A 10-min warm-up and 5-min cool-down with low intensity were incorporated at the commencement and conclusion of each training session^63^.

### Anthropometric measurements

The height of the animal was measured by nose-anus length using centimeters in anesthetized rats, and weight using a precise digital scale (Jadever Scale Co.) with an accuracy of 0.1 g. Body mass index was determined by dividing the weight(gr) and the square of the nose-anal length (cm)^64^. These parameters were measured for all rats weekly, between 9:00 a.m. and 11:30 a.m. Also, waist circumference (WC) was measured in the largest region of the rat abdomen by a flexible tape with an accuracy of 0.1 cm^31^.

### Biochemical measurements

Rats were sacrificed after deep anesthesia by intraperitoneal injection of ketamine (60 mg/kg) and xylazine (5 mg/kg) in a ratio of 4:1. Then their abdomen were opened by a midline incision made on skin, and abdominal muscle, and immediately 7 ml blood was taken from inferior vena cava with a syringe, and then liver samples were collected. Remaining corpses were transferred to animals freezer.

Then serum was separated by rapid centrifugation (3000 rpm for 15 min), and stored together with liver samples at -80 °C for subsequent biochemical measurements.

### Enzyme-linked Immunsorbent assay (ELISA assay)

The serum asprosin, TNF-α, and insulin concentrations were determined using the rat (Asprosin ELISA KIT (Catalog Number: MBS1600686, Aviscera Bioscience, Inc., United States) (RAT TNF-α, ELISA kit, Catalog Number: MBS1600686, Bio-Techne, United States) and (insulin ELISA kit, Catalog Number: 80-INSRTH-E01, E10, MyBioSource, United States) respectively.

### Colorimetry

Glucose concentration was measured using the glucose oxidase method with a spatial kit (catalog number P.L:64780179) from Pars Azmoun Company, which has a sensitivity of 5 mg/dL. Lipid profiles (TC, TG) were determined using (Pars Azmoun Company kits, catalog number P.L:38231049, P.L:38231049), while HDL and LDL were measured using colorimetry kits (Darman Faraz Kave Company, catalog number P.L:146, P.L:157).

Finally, the insulin resistance index (HOMA-IR) was calculated using the homeostasis method based on the following formula: ${\text{ HOMA-IR}} = \left[ {{\text{fasting glucose }}\left( {{\text{mmol}}/{\text{L}}} \right){ } \times {\text{ fasting insulin }}\left( {{\text{U}}/{\text{mL}}} \right)} \right]{ } \div { }5.22$.

Quantitative insulin-sensitivity check index (QUICKI)^37^ was calculated as 1/ [log (G0) + log (I0)], where G0 is fasting glycaemia (mg dl^−1^), and I0 is fasting insulin level (U ml^−1^)^65^.

MetS severity Z-score was calculated by the following formula : ${\text{MetS severity Z-score:}}\left[ {\left( {{\text{HDL}}_{ = } 37} \right){ }/8.3} \right] + \left[ {\left( {{\text{LDL}}_{ = } 22} \right){ }/3.9} \right] + \left[ {\left( {{\text{TG}}_{ = } 42} \right){ }/7.1} \right] + \left[ {\left( {{\text{fasting glucose}}_{ = } 130} \right){ }/15.5} \right] + \left[ {\left( {{\text{insulin}}_{ = } { }7} \right){ }/2.6} \right] + \left[ {\left( {{\text{WC}}_{ = } { }17} \right){ }/1.14} \right]$

### Western blotting

Five gr liver tissue was homogenized using a lysis buffer and then the homogenized mixture was centrifuged for 10 min at 12,000 rpm at 4 °C. The obtained supernatants were separated on a 15% sodium dodecyl sulfate–polyacrylamide gel electrophoresis (SDS-PAGE) and transferred into a polyvinylidene difluoride (PVDF) membrane using a transfer buffer. The PVDF membrane was blocked with 2% skim milk in TBS-T buffer for 75 min and was incubated for 16–18 h at room temperature with diluted (1/300) primary antibodies of [anti-β-actin sc-47,778, Santa Cruz Biotechnology, USA, anti-AMPK (AMPKα1/2 (D-6), sc-74461, Santa Cruz Biotechnology, USA, anti-p-AMPK (p-AMPKα1/2 (Thr 172), sc-33524Santa Cruz Biotechnology, USA), anti- asprosin, FNab09797, Fine Biotech Co., Ltd., China at dilution of 1:400). After washing 3 times with TBS-T buffer, it was incubated with diluted secondary antibody m-IgGκR BP-HRP (Santa Cruz Biotechnology, USA, sc-516,102) and 1/1000 rat anti-rabbit IgG-HRP (Santa Cruz Biotechnology, USA, sc-2357), for 75 min at room temperature. Then washing procedure was repeated and finally, the PVDF was incubated with substrate solution for 1–2 h at room temperature to give the band^66^. Digital images generated by a document scanner were used to quantify total proteins on the blots in Image J software. Beta-actin and saline groups were used as normalizing protein and control groups respectively for the analysis of western blot.

### Statistical analysis

The normality of data was evaluated by the Shapiro–Wilk test; then then independent t-test, one-way ANOVA, and Tukey's method were used in SPSS 25.0 program (SPSS, Inc., Chicago, IL, USA). P values less than 0.05 were considered significant and Graphs were drawn using Graph Pad Prism version 9 software. All results are shown in mean ± standard error. Values of *P* < 0.05 were considered statistically significant.

### Supplementary Information

Supplementary Information.

## Data Availability

The results of the current study are available from the corresponding author on reasonable request.
