# Supplementary material for: Comparison of HIIT and MICT and further detraining on metabolic syndrome and asprosin signaling pathway in metabolic syndrome model of rats
Source: Sci Rep. 2024 May 17;14:11313. doi: 10.1038/s41598-024-61842-5 (PMC11101655; doi:10.1038/s41598-024-61842-5)
Supplement: Supplementary file 1 — Supplementary Information. [file 41598_2024_61842_MOESM1_ESM.pdf]

Comparison of HIIT and MICT and further detraining on metabolic syndrome and asprosin signaling pathway in metabolic syndrome model of rats

Hiwa Ahmed Rahim [1, 2, 4], Arsalan Damirchi1, Parvin Babaei [3, 4, 5*]

1- Department of Exercise Physiology, Faculty of Sport Sciences, University of Guilan, Rasht, Iran

2-College of Physical Education and Sports Sciences, University of Halabja, Halabja, Iraq

3- Neuroscience Research Center, School of Medicine, Guilan University of Medical Sciences, Rasht, Iran

4-Cellular & Molecular Research Center, School of Medicine, Guilan University of Medical Sciences, Rasht, Iran

5- Department of Physiology, School of Medicine, Guilan University of Medical Sciences, Rasht, Iran

*Correspondent author:

Dr Parvin Babaei

Tel/Fax: +98 9113313747

Email: p_babaei@gums.ac.ir

https://orcid.org/ **0000-0003-4870-8182**

Neuroscience Research Center, School of Medicine, Guilan University of Medical Sciences, Rasht, Iran.

Original blots supplementary files.

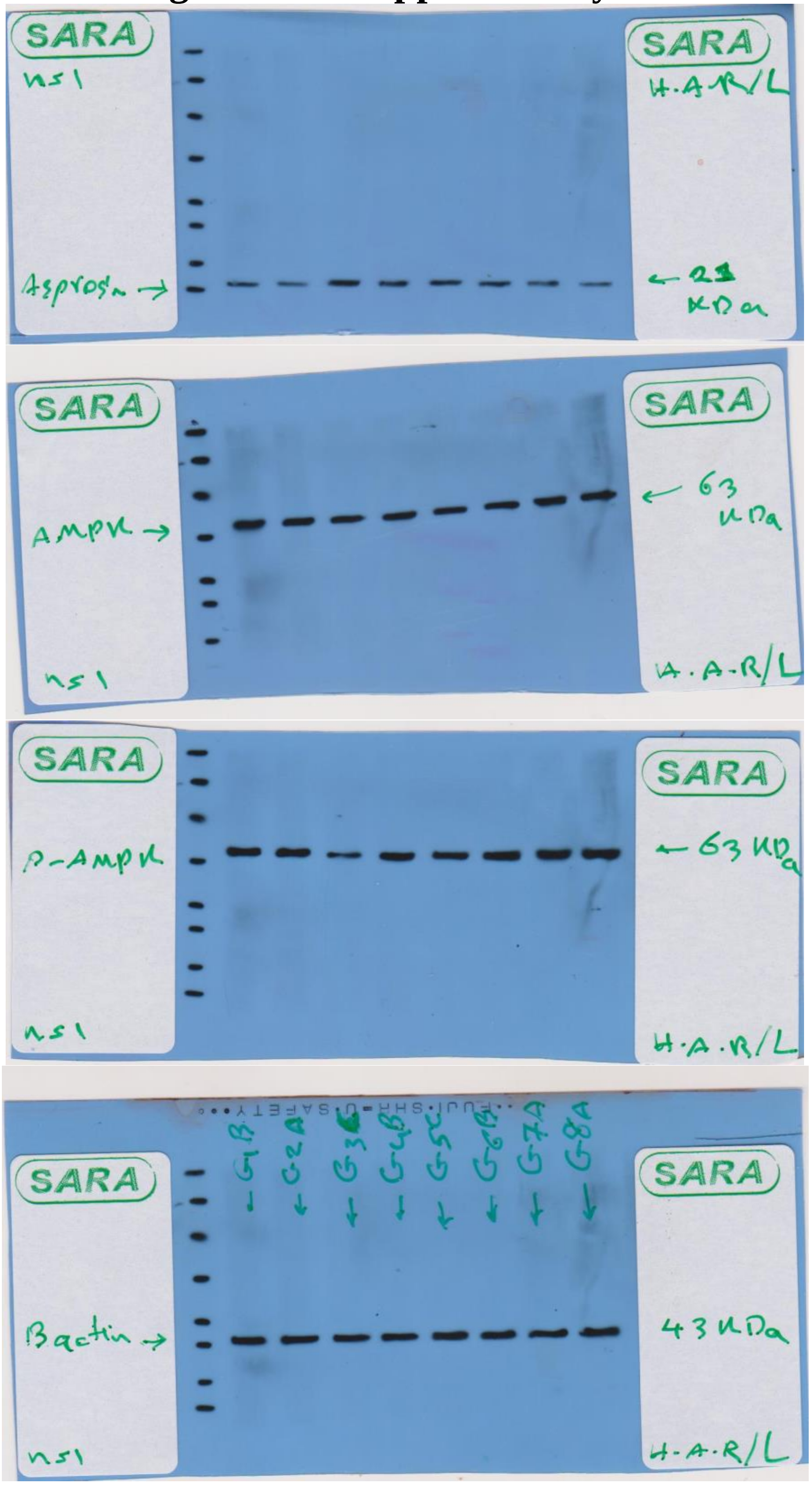

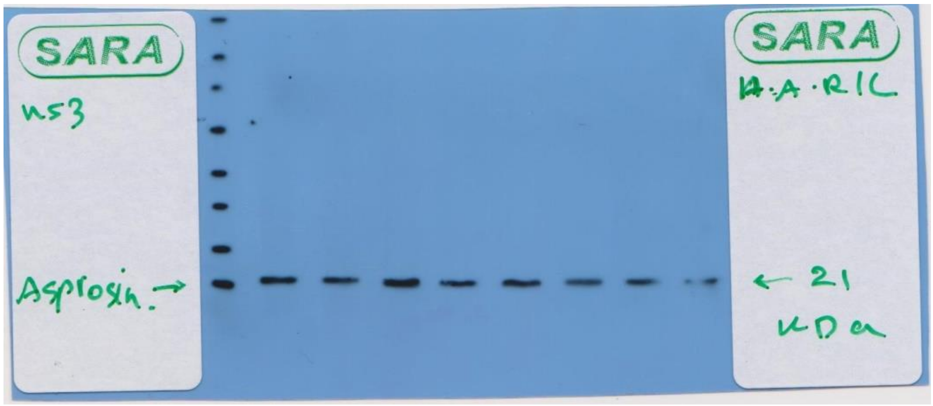
SARA
ns3
Asprosin →
SARA
H.A.R/L
← 21
kDa

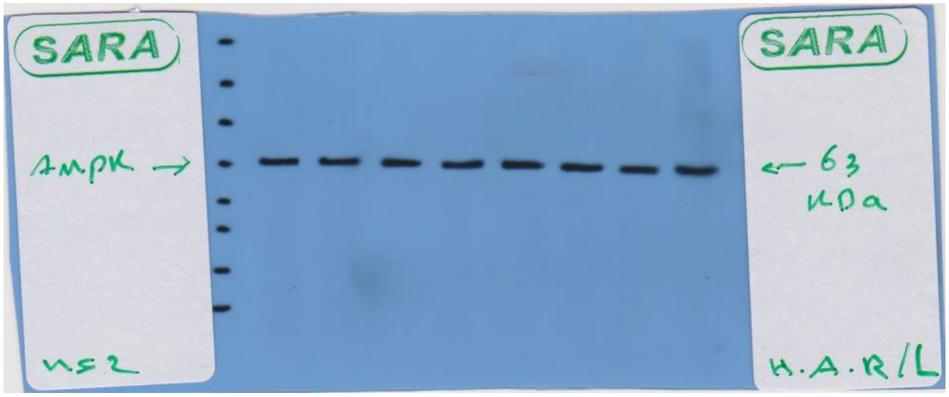
SARA
AMPK →
ns2
SARA
← 63
kDa
H.A.R/L

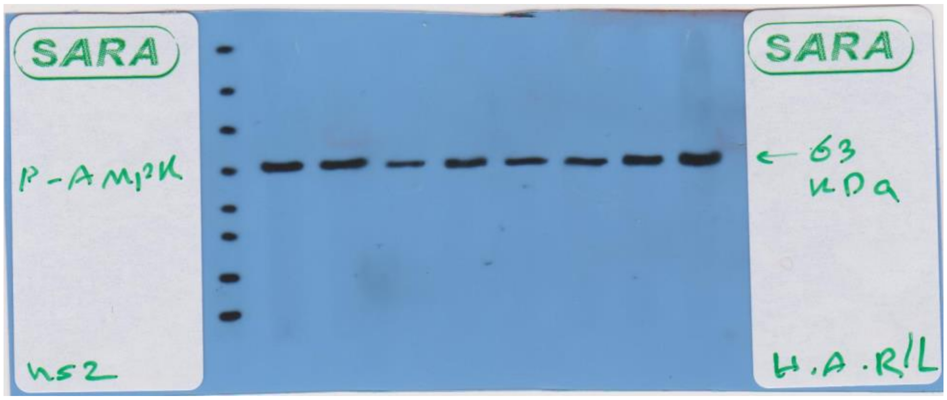
SARA
P-AMPK
ns2
SARA
← 63
kDa
H.A.R/L

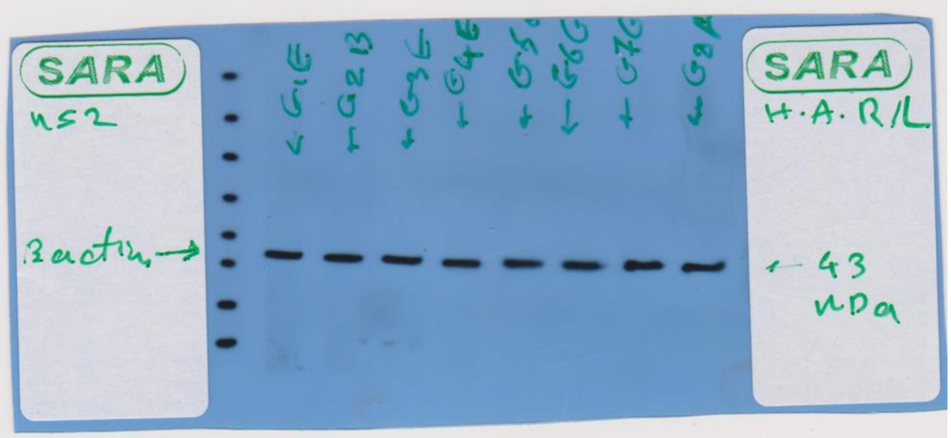
SARA
ns2
β actin →
SARA
H.A.R/L
← 43
kDa

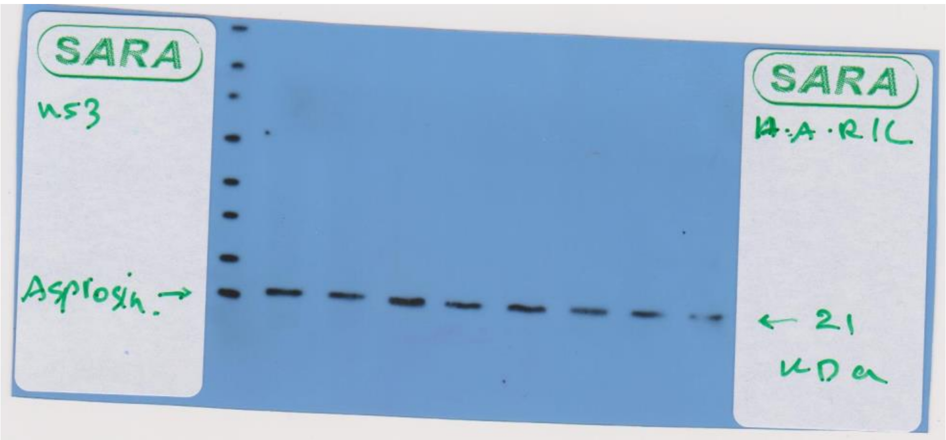
SARA
ns3
Asprosin →
SARA
H.A.R/L
← 21 kDa

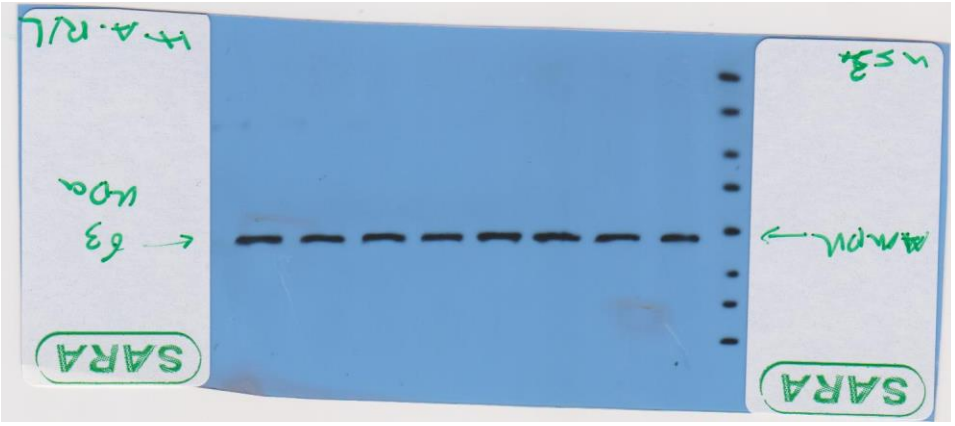
H.A.R/L
ns3
63 → kDa
AMPK ←
SARA
SARA

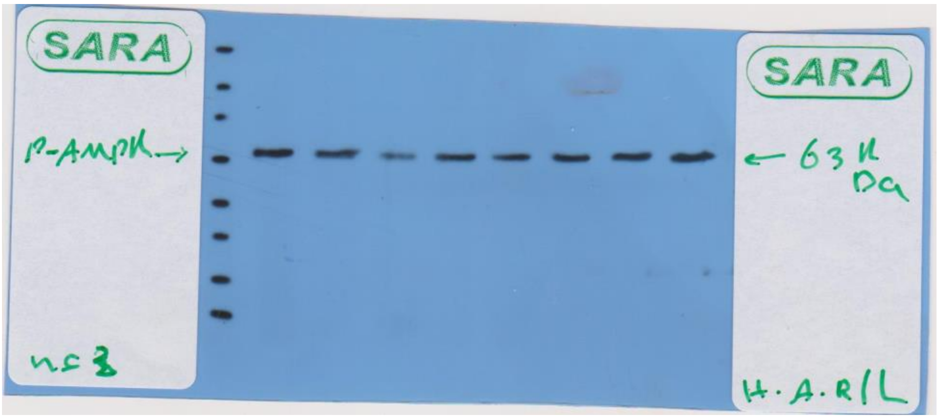
SARA
P-AMPK →
ns3
SARA
← 63 kDa
H.A.R/L

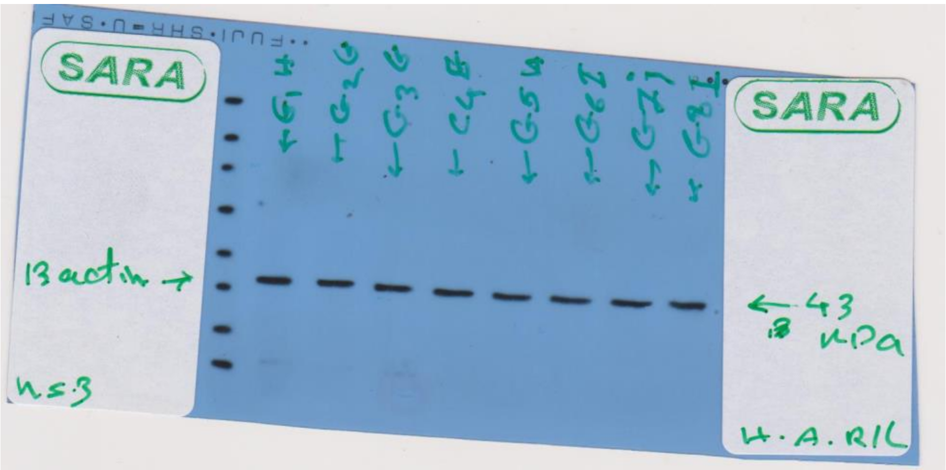
SARA
B actin →
ns3
SARA
← 43 kDa
H.A.R/L
